# Is chronic fatigue syndrome truly associated with haplogroups or mtDNA single nucleotide polymorphisms?

**DOI:** 10.1186/s12967-016-0939-0

**Published:** 2016-06-18

**Authors:** Josef Finsterer, Sinda Zarrouk-Mahjoub

**Affiliations:** Krankenanstalt Rudolfstiftung, Postfach 20, 1180 Vienna, Austria; Genomics Platform, Pasteur Institute of Tunis, Tunis, Tunisia

**Keywords:** Mitochondrial DNA, Chronic fatigue syndrome, Exercise intolerance, Exhaustion, Respiratory chain

**Letter to the Editor**

With interest we read the article by Billing-Ross et al. [1] about 193 patients with chronic fatigue syndrome (CFS) diagnosed according to the Fukuda or Canadian Consensus criteria and undergoing sequencing of the mtDNA, the DePaul Symptom questionnaire and the Medical Outcome Survey Short Form-36. The study showed that CFS is associated with mtDNA haplogroups J, U and H, that 8 mtDNA single nucleotide polymorphisms (SNPs) were associated with 16 symptom categories, and that three haplogroups were associated with six symptom categories [1]. We have the following comments and concerns.

The main limitation of this study is that only the mtDNA was investigated for sequence variants. Since it is well-known that mitochondrial disorders (MIDs) may be also caused by mutations in nDNA-located genes, particularly in children [2], disease-causing mutations or SNPs facilitating the development of CFS may have been missed. Furthermore, MIDs may not only be due to respiratory chain dysfunction but also due to disruption of other mitochondrial pathways, such as the beta-oxidation, the hem synthesis, the calcium handling, the coenzyme-Q metabolism, or the urea cycle. There is also consensus that investigations of mtDNA mutations or SNPs in mtDNA from lymphocytes may not be constructive since some mutations may not be present or heteroplasmy rates may be lower than in more severely affected tissues [3].

A further limitation of the study is that neither immune-histological nor biochemical investigations of the muscle biopsy were carried out. Immune-histological investigations of the muscle biopsy may show NADH-, SDH-, or COX-deficiency. Biochemical investigations of the muscle homogenate may show reduced activity of one or several respiratory chain complexes or coenzyme-Q deficiency [4]. Morphological and functional studies are essential not to miss dysfunction of the respiratory chain or other mitochondrial pathways.

We also should be informed how causes of fatigue, exhaustion, and exercise intolerance other than CFS, were excluded. How many patients had muscle disease other than a MID, which may be also associated with fatigue, such as muscular dystrophies or congenital myopathies [5, 6]? How many had hypothyroidism, sleep apnea syndrome, malignancy, electrolyte disturbances, adrenal dysfunction, or pituitary insufficiency? Were any of the routine laboratory parameters abnormal in the 193 patients? How many had creatine-kinase (CK) elevation or lactacidosis? How many presented with features other than fatigue which could be attributed to a MID? How many had epilepsy, cognitive impairment, extra-pyramidal disease, or psychiatric disease? How many had a cataract, glaucoma, retinitis, or chronic progressive external ophthalmoplegia (CPEO)? It would be interesting to known if any of the patients had cardiac disease, gastrointestinal disease, renal impairment, endocrine disturbances, or lung disease indicative of a MID?

Nothing is reported about the previous history of the 197 patients and their permanent medication [1]. How often was the family history positive for CFS or a MID?

Overall, this interesting study may profit from providing more information about the individual patients and their relatives and from carrying out more comprehensive morphological, functional, and genetic studies not to miss metabolic defects, which may be due to sequence variations in nDNA located mitochondrial genes. There is also a need to exclude cardiac, pulmonary, or systemic disease not to miss differentials of CSF.
